# Diagnostic value of SPECT, PET and PET/CT in the diagnosis of coronary artery disease: A systematic review

**DOI:** 10.2349/biij.7.2.e9

**Published:** 2011-04-01

**Authors:** M Al Moudi, Z Sun, N Lenzo

**Affiliations:** 1 Discipline of Medical Imaging, Curtin University of Technology, Perth, Western Australia, Australia; 2 Department of Nuclear Medicine, Fremantle Hospital, Perth, Western Australia, Australia

**Keywords:** Coronary artery disease, single photon emission computed tomography, positron emission tomography, computed tomography, diagnostic value

## Abstract

**Purpose:**

The purpose of the study was to investigate the diagnostic value of SPECT, PET and PET/CT in the diagnosis of coronary artery disease, based on a systematic review.

**Material and Methods:**

A search of PubMed/Medline and Sciencedirect databases in the English-language literature published over the last 24 years was performed. Only studies with at least 10 patients comparing SPECT, PET or combined PET/CT with invasive coronary angiography in the diagnosis of coronary artery disease (50% stenosis) were included for analysis. Sensitivities and specificities estimates pooled across studies were analysed using a Chi-square test.

**Results:**

Twenty-five studies met the selection criteria and were included for the analysis. Ten studies were performed with SPECT alone; while another six studies were performed with PET alone. Five studies were carried out with both PET and SPECT modalities, and the remaining four studies were investigated with integrated PET-CT. The mean value of sensitivity, specificity and accuracy of these imaging modalities for the diagnosis of coronary artery disease was 82% (95%CI: 76 to 88), 76% (95%CI: 70 to 82) and 83% (95%CI: 77 to 89) for SPECT; 91% (95%CI: 85 to 97), 89% (95%CI: 83 to 95) and 89% (95%CI: 83 to 95) for PET; and 85% (95%CI: 79 to 90), 83% (95%CI: 77 to 89) and 88% (95%CI: 82 to 94) for PET/CT, respectively. The diagnostic accuracy of these imaging modalities was dependent on the radiotracers used in these studies, with ammonia resulting in the highest diagnostic value.

**Conclusion:**

Our review shows that PET has high diagnostic value for diagnosing coronary artery disease, and this indicates that it is a valuable technique for both detection and prediction of coronary artery disease.

## INTRODUCTION

Coronary artery disease (CAD) remains the leading cause of mortality and morbidity in Western countries [1]. Invasive coronary angiography is currently the gold standard for diagnosis and treatment of CAD; however, it is an invasive procedure associated with risks and complications [2]. Moreover, it is reported that around 20% to 40% of all diagnostic invasive coronary angiography procedures were performed for diagnostic purposes without any interventional procedures being applied [3–5]. Thus, investigation of less invasive imaging modalities is important for reducing or avoiding the use of invasive coronary angiography examinations [4].

Currently, multislice computed tomography (CT) angiography is widely used in clinical practice for the diagnosis of CAD, and its diagnostic accuracy has been significantly enhanced with the recent development of 64-, 256- and 320-slice scanners [6–9]. Studies have shown that multislice CT angiography can be used as a reliable alternative to invasive coronary angiography in selected patients, due to its high sensitivity and specificity [6–9].

Myocardial perfusion imaging with SPECT is a widely established method for non-invasive evaluation of coronary artery stenosis [10]. However, the most important applications of SPECT are in the diagnosis of CAD, prediction of disease prognosis, selection of patients for revascularisation and assessment of acute coronary syndromes. Moreover, SPECT holds special value in some particular patient subgroups [11, 12]. Generally speaking, the sensitivity of stress SPECT for detecting angiographically-defined CAD is consistently above 70%, but in the better-designed studies, it is within the range of 85–90% [13, 14].

Positron emission tomography (PET) has contributed significantly to advancing our understanding of heart physiology and pathophysiology for more than 25 years. The diagnostic accuracy of myocardial perfusion by PET in the assessment of CAD has been reported to be superior to SPECT [15, 16]. PET with rest-stress myocardial perfusion is regarded as an exact imaging modality for diagnosing and managing patients with CAD [16]. Moreover, the combined modality of PET/CT further increases the diagnostic accuracy in CAD [12–16].

Despite promising results reported in the literature [17, 18], the diagnostic value of SPECT and PET to detect CAD has not been well established. This is mainly due to the fact that the diagnostic accuracy reported by these studies is variable and the radiopharmaceuticals used in these studies are different. Thus, the purpose of our study was to investigate the diagnostic value of SPECT, PET and PET/CT when compared to invasive coronary angiography for detection of CAD, based on a systematic review of the current literature.

## MATERIALS AND METHODS

A search of the English-language literature was performed using two main databases, PubMed/Medline and ScienceDirect. The search included articles published between 1985 and 2009 on the topics of SPECT, PET and PET/CT in CAD. The research was limited to peer-reviewed articles on human subjects and studies published in the English language. The keywords used for the search were “Positron Emission Tomography”, “Single Photon Emission Computed Tomography”, “integrated Positron Emission Tomography and Computed Tomography”, “Coronary Artery Disease”, “Myocardial perfusion”, “Nuclear Medicine Imaging in cardiac disease”. The reference lists for studies matching these criteria were also reviewed to identify additional articles which were not found through the initial search. The last search was performed in September 2010 to ensure inclusion of all recent publications in the analysis.

Articles that met the following criteria were included for analysis: SPECT, PET, or PET/CT studies were performed in patients who underwent myocardial perfusion imaging (MPI) rest/stress test while invasive coronary angiography was used as the standard of reference; at least ten patients were included in each study; diagnosis of CAD was based on >50% stenosis. The reason >50% stenosis was chosen was because it has become a routine clinical practice to consider coronary stenosis >50% as haemodynamically significant [19–21]. Also, the diagnostic value in terms of sensitivity and specificity was reported in the study. The exclusion criteria included: review articles or case study reports; animal or phantom studies; studies dealing with myocardial perfusion without addressing the diagnostic accuracy of coronary artery stenosis or occlusion, and studies including patients treated with coronary stents or bypass grafts.

Two reviewers checked the references independently and the following information was extracted from each study: year of publication; number of participants in the study; prevalence of patients with suspected or confirmed CAD; mean age; percentage of male patients affected; type of radiotracer used in each study; rest and stress imaging protocols; diagnostic value of SPECT, PET and PET/CT in terms of sensitivity and specificity; and the accuracy of SPECT, PET and PET/CT for detection of CAD. The main findings were summarised in terms of the extent to which studies were reported to have shown the value of using SPECT, PET and PET-CT to diagnose CAD towards improving patient management and cost-effectiveness.

All of the statistical analyses were undertaken using SPSS software version 17.0 (SPSS Inc., Chicago, ILL). Each of the study estimates for sensitivity and specificity were independently combined across studies using one sample test. Comparison was performed by Chi Square test using n-1 degree of freedom to test if there are any significant differences between different imaging modalities (SPECT vs PET, SEPCT vs PET/CT, PET vs PET/CT). Statistical hypotheses (2-tailed) were tested at the 5% level of significance.

## RESULTS

Twenty-five studies (with 25 comparisons) met the selection criteria and were included in the analysis [19–43]. Ten of these studies were performed with SPECT alone [19–24, 39–41, 43], while six of the studies were performed with PET alone [30–35]. Five studies were carried out with both PET and SPECT modalities [25–29] and the remaining four studies were investigated with integrated PET/CT [36–38, 42]. The total number of patients included in these studies was 4,114 with 53.5% of the patients suspected of CAD, 72.2 % being male and the mean age of patients being 48-years-old. Table 1 summarises the number of patients, the radiotracers used in each study, and the reported sensitivity, specificity and accuracy with use of SPECT, PET and PET/CT imaging modalities, respectively.

**Table 1:** Study characteristics of SPECT, PET and PET/CT for detection of coronary artery disease

**Authors**	**Year**	**Patients (n)**	**Male (%)**	**Mean age (yr)**	**Stenosis**	**Radiotracer**	**Sensitivity**	**Specificity**	**Accuracy**
Husmann et al^16^	2008	80	85	36	>50%	^201^Thallium	77%	84%	NA
Fallahi et al^20^	2008	51	85	34	NA	^99m^Tc-sestamibi	91%	71%	88%
Di Carli et al^35^	2007	110	54.5	57.3	>50%	^82^Rubidium	65%	75%	NA
Cesar et al^36^	2007	281	51.6	38.3	>50%	^82^Rubidium	93%	75%	91%
Sampson et al^37^	2007	102	59	37	>50%	^82^Rubidium	93%	83%	85%
Bateman et al^11^	2006	85	52	65	>50%	^99m^Tc-sestamibi	82%	73%	79%
Elhendy et al^21^	2001	332	77	57	>50%	^99m^Tc-sestamibi	77%	74%	77%
Leoncini et al^22^	2001	33	93	37	NA	^99m^Tc-sestamibi	85%	55%	NA
Nakamura et al^23^	1999	81	80	37	>50%	^201^Thallium & ^99m^Tc-sestamibi	83%	99%	95%
Levine et al^24^	1999	50	76	36	NA	^99m^Tc-sestamibi	86%	55%	85%
Milavetz et al^25^	1998	209	77	36.5	>50%	^99m^Tc-sestamibi	95%	73%	88%
Williams et al^29^	1994	287	75	NA	>50%	^82^Rubidium	87%	88%	88%
Simone et al^30^	1992	225	80	NA	>50%	^82^Rubidium	92%	91%	91%
Marwick et al^31^	1992	74	NA	34	>50%	^82^Rubidium	90%	100%	91%
Grover-McKay et al^32^	1992	31	84	34.5	>50%	^82^Rubidium	100%	73%	87%
Stewart et al^26^	1991	81	64.3	35	>50%	^201^Thallium	87%	53%	78%
Go et al^27^	1990	202	NA	NA	>50%	^201^Thallium	76%	80%	77%
Demer et al^33^	1989	193	74.1	NA	>50%	^82^Rubidium & ^13^N ammonia	83%	95%	85%
Tamaki et al^28^	1988	51	NA	56.1	>50%	^201^Thallium	81%	92%	NA
Gould et al^34^	1986	50	NA	NA	>50%	^82^Rubidium & ^13^N ammonia	95%	100%	97%

NA-not available

The degree of coronary artery stenosis was assessed based on the criterion of >50%, which was determined by invasive coronary angiography in all studies. Of these 20 studies, 82Rubidium was the most commonly-used radiopharmaceutical which was utilised in 10 studies [26–28, 30–33, 36–38]; 99mTc-Sestamibi was used in seven studies [19–21, 23, 24, 26, 41]; 201Thallium was used in four studies [25, 26, 28, 29]; a combination of 201Thallium and 99mTc-Sestamibi was used in one study [22]; 99mTc-Tetrofosmin was used in three studies [39, 40, 43]; and combinations of 13N Ammonia and 82Rubidium were used in the remaining two studies [34, 35].

### Analysis of diagnostic value of SPECT in CAD

A total of 2,208 patients were included in 15 SPECT studies based on significant CAD which was determined by visual assessment of coronary angiography. Figure 2 shows the mean sensitivity, specificity and accuracy of SPECT for diagnosis of CAD. As shown in the figure, the diagnostic value of SPECT was moderate when compared to invasive coronary angiography.

**Figure 1:**
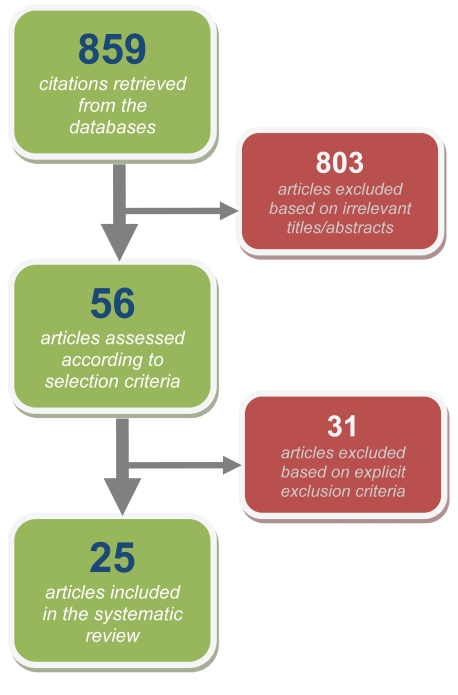
Flow chart shows the searching strategy of eligible references.

**Figure 2:**
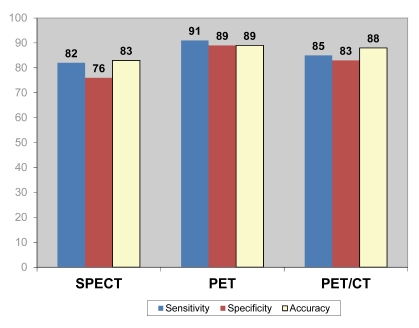
Overall pooled mean diagnostic value of SPECT, PET and PET/CT for detection of coronary artery disease.

### Analysis of diagnostic value of PET in CAD

1,376 patients were included in another 11 studies performed with PET tests based on significant CAD which was determined by visual assessment of coronary angiography. Figure 2 shows the mean sensitivity, specificity and accuracy of PET for diagnosis of CAD. As shown in the figure, PET imaging was found to have high diagnostic value in the detection of CAD.

### Analysis of diagnostic value of PET/CT in CAD

518 patients were included in four studies performed with PET/CT studies based on significant CAD which was determined by visual assessment of coronary angiography. Again Figure 2 demonstrates that moderate sensitivity, specificity and accuracy of SPECT were reached for diagnosis of CAD.

### Comparison of SPECT, PET and PET/CT

There is a significant difference in sensitivity, specificity and accuracy between PET and SPECT, PET and PET/CT, SPECT and PET/CT for the diagnosis of CAD (p < 0.05), with PET demonstrating the highest diagnostic value among these three imaging modalities as shown in Figure 2.

### Analysis of diagnostic value for assessment of individual coronary artery disease

Table 2 demonstrates the diagnostic value of SPECT, PET and PET/CT in three studies with five comparisons based on analysis of three main coronary arteries.

**Table 2:** Mean diagnostic value of SPECT, PET and PET/CT for detection of coronary artery disease by individual coronary branches.

**Individual vessel CAD using 50% stenosis**	**Radiotracer**			**Sensitivity**			**Specificity**			**Accuracy**		
	**SPECT**	**PET**	**PET/CT**	**SPECT**	**PET**	**PET/CT**	**SPECT**	**PET**	**PET/CT**	**SPECT**	**PET**	**PET/CT**
Bateman et al^11^ >50% stenosis	^99m^Tc-sestamibi/ ^82^Rubidium	^99m^Tc-sestamibi/ ^82^Rubidium										
LAD				61%	79%		92%	95%		75%	87%	
LCx				33%	58%		86%	93%		68%	79%	
RCA				60%	58%		87%	100%		73%	78%	
Tamaki et al^28^ >50% stenosis	^201^Thallium/ ^13^N ammonia	^201^Thallium/ ^13^N ammonia								NA	NA	
LAD				90%	93%		89%	100%				
LCx				65%	85%		93%	90%				
RCA				79%	76%		95%	86%				
Cesar et al^36^ >50% stenosis			^82^Rubidium									
LAD						70%			69%			70%
LCx						76%			86%			83%
RCA						66%			92%			77%

**Figure 3:**
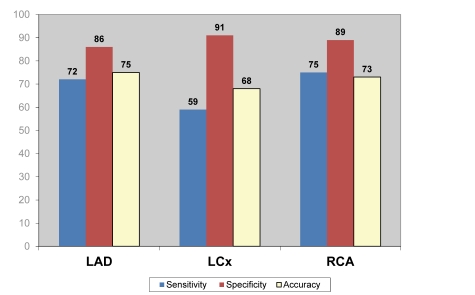
Pooled diagnostic value of SPECT for detection of coronary artery disease based on individual coronary artery assessment.

**Figure 4:**
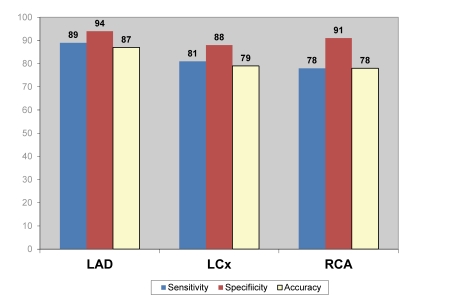
Pooled diagnostic value of PET for detection of coronary artery disease based on individual coronary artery assessment.

**Figure 5:**
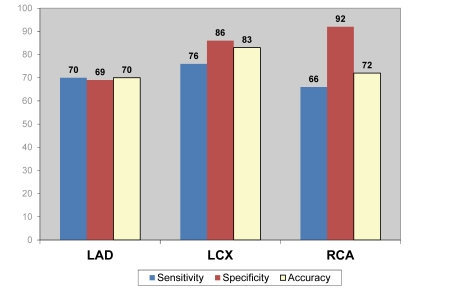
Pooled diagnostic value of PET/CT for detection of coronary artery disease based on individual coronary artery assessment.

Figures 3 to 5 demonstrate the overall results of sensitivity, specificity and accuracy analysed with three different nuclear medicine modalities at left anterior descending (LAD), left circumflex (LCx) and right coronary artery (RCA). As shown in the figures, PET has the highest diagnostic value among the 3 imaging modalities for diagnosis of CAD.

### Analysis of effects of different Radiotracers on diagnostic value

Since different types of radiotracers were used in these 20 studies, analyses of the diagnostic value of variable radiotracers for detection of CAD were investigated. Table 3 shows the radiotracers used in these studies and their corresponding diagnostic value. As demonstrated in the table, using ammonia as a radiotracer produced the highest diagnostic value for detection of CAD.

**Table 3:** Mean diagnostic value of nuclear medicine imaging with use of different radiotracers.

**Radiotracer**	**Patient**	**Male**	**Mean age**	**Stenosis**	**Sensitivity**	**Specificity**	**Accuracy**
^201^Thallium	414	74.6	42.3	50%	80%(95% CI: 74 to 86 )	77%(95% CI: 71 to 83 )	78%(95% CI: 72 to 84 )
^99m^Tc-sestamibi	760	76.6	45.7	50%	86%(95% CI: 80 to 92 )	66%(95% CI: 60 to 72 )	83%(95% CI: 77 to 89 )
^201^Thallium & ^99m^Tc-sestamibi	81	80	37	50%	83%(95% CI: 77 to 89 )	99%(95% CI: 93 to 100 )	95%
^13^N ammonia	121	71	42.5	50%	92%(95% CI: 86 to 98 )	86%(95% CI: 80 to 92 )	90%(95% CI: 84 to 96 )
^82^Rubidium	985	71	42.5	50%	90%(95% CI: 84 to 96 )	88%(95% CI: 82 to 94 )	92%(95% CI: 86 to 98 )
^82^Rubidium & ^13^N ammonia	243	74	NA	50%	89%(95% CI: 83 to 95)	97%(95% CI: 91 to 100 )	91%(95% CI: 75 to 87 )

**Figure 6:**
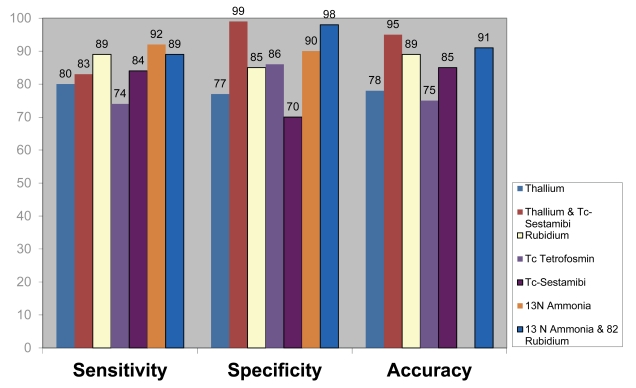
Pooled diagnostic value of nuclear medicine imaging with use of variable radiotracers for detection of coronary artery disease.

Figure 6 shows the differences in sensitivity and specificity between both 201Thallium and 99mTc-sestamibi for the diagnosis of CAD (p < 0.05), and also significant differences in the specificity and accuracy between both 201Thallium and 99mTc-sestamibi and individual radiotracers for the diagnosis of CAD (p < 0.05).

Significant differences in sensitivity and accuracy was found between 13N ammonia, 82Rubidium and both 13N ammonia with 82Rubidium for the diagnosis of CAD (p < 0.05) as shown in Figure 6. Significant differences were found in the sensitivity and specificity between use of combined radiotracers (13N ammonia and 82Rubidium, 201Thallium and 99mTc-sestamibi) and use of individual radiotracers alone for the diagnosis of CAD (p < 0.05).

## DISCUSSION

This systematic review presents three significant findings which are considered to be important from a clinical perspective. First, the diagnostic value of SPECT and PET/CT in the detection of CAD is moderate when compared to invasive coronary angiography. Second, PET has higher sensitivity and specificity than SPECT or PET/CT for detection of CAD, indicating the increasing diagnostic accuracy of PET in cardiac imaging. Lastly, there are significant differences when different radiotracers are used for the diagnosis of CAD, with the use of combined radiotracers resulting in improved diagnostic value.

SPECT has been used as a routine technique in clinical practice for myocardial perfusion imaging for decades [28]. Previous studies have shown that the diagnostic value of SPECT in cardiac imaging is variable, ranging from low to moderate [29]. Di Carli et al. [44] compared three studies using SPECT with PET, looking particularly at the diagnostic accuracy for detection of CAD. Only the sensitivity and specificity were provided in their study while the analysis of diagnostic accuracy was not identified. This analysis confirms their results to a greater extent, as the mean sensitivity, specificity and accuracy of SPECT for detection of CAD are moderate. This indicates that SPECT has not reached the diagnostic accuracy to be considered as a reliable technique for assessment of CAD.

PET has been used more recently in cardiac imaging, and early results look promising [45]. PET offers potential advantages in a clinical practice over myocardial perfusion scintigraphy [46]. PET is able to assess myocardial blood flow and is superior in detecting multivessel disease [47]. Our analysis identifies PET as having the highest diagnostic value for CAD among the three different nuclear imaging modalities analysed. Husmann et al. [26] compared SPECT and PET in respect to diagnostic accuracy of myocardial perfusion imaging. Only the sensitivity and specificity were reported in their study, while the diagnostic accuracy was not available. In contrast, the sensitivity, specificity and accuracy of PET for detection of CAD were analysed in our report. This review provides a comprehensive analysis of the diagnostic value of these nuclear modalities, including sensitivity, specificity and accuracy. Thus, it is believed that the analysis offers additional and valuable diagnostic information, as compared to the previous reports in the literature.

Recently, cardiac imaging has been further enhanced by the use of integrated PET/CT, a combined modality which provides the considerable benefit of anatomical and physiologic assessment in patients with CAD [36, 37]. PET/CT allows precise detection and localisation of CAD [38]. CT coronary angiography provides superior anatomical details but lacks the functional information of cardiac perfusion. The latest refinements in CT technology, including multidetector CT with faster gantry rotations, and dual-source devices, have advanced CT angiography as a promising alternative to conventional angiography in the diagnosis of CAD selected patients [48]. Recent studies have shown that 64-slice CTA has high sensitivity (73%- 100%) and specificity (90%-90%) in the detection of CAD [49–51]. PET offers evidence of sub-clinical coronary atherosclerosis as it is superior in demonstrating metabolic activities, but its spatial resolution is limited when demonstrating coronary anatomical structures [47]. Thus, combined PET/CT overcomes the limitations of each individual modality while maximising the advantages of both PET and CT in cardiac imaging [44–46].

However, the analysis of integrated PET/CT for detection of CAD was not as good as initially expected since PET/CT was shown to have moderate sensitivity, specificity and accuracy. This may be due to the selection of patients with different risk factors in the studies analysed. Therefore, results of this analysis should be interpreted with caution. PET/CT may show improved diagnostic accuracy in other areas, such as tumour imaging, but not in the diagnosis of CAD, based on this analysis.

This analysis also involves a comparison of radiotracers used in SPECT and PET imaging which includes seven different types of radioisotopes, namely 201Thallium, 99mTc-tetrofosmin, 99mTc-Sestamibi, a combination of 201Thallium and 99mTc-Sestamibi, 13N Ammonia, 82Rubidium, and a combination of 13N Ammonia and 82Rubidium. The comparative analysis indicates significant benefits of using 13N ammonia for MPI to detect CAD by PET, leading to the highest sensitivity and accuracy. Both 13N ammonia and 82Rubidium have significantly high specificity in MPI by PET. PET imaging with use of 13N ammonia, 82Rubidium, and a combination of 13N ammonia and 82Rubidium for diagnosis of CAD, has been found to result in significant differences in sensitivity, specificity and accuracy than those using 201Thallium, 99mTc-sestamibi, and a combination of 201Thallium and 99mTc-sestamibi by SPECT imaging. This is consistent with results reported by other studies. Go and colleagues [28] compared PET and SPECT in 202 patients. Their results showed there was no significant difference between PET imaging with use of 82Rubidium and SPECT imaging with 201Thallium. Tamaki and colleagues [29] compared 13N ammonia PET with 201Thallium SPECT and reported similar findings. Hence, this analysis confirms that 13N ammonia PET and 201Thallium SPECT provide high diagnostic value for detection of CAD.

In addition, the analysis of individual coronary arteries demonstrates the superiority of PET over the other two modalities, but a significant difference in diagnostic value was only found between PET and SPECT, PET/CT in LAD. PET/CT was found to be significantly higher than PET in the assessment of RCA in terms of diagnostic accuracy. However, only a few studies presented the analysis of individual coronary arteries, thus a robust conclusion cannot be drawn based on the current analyses.

There were some limitations identified in this study which need to be addressed. First, most of the studies did not provide detailed numbers of true positive, true negative cases, thus this prevented the authors from performing an accurate analysis of the diagnostic value of these modalities. Second, studies currently under review or submitted for publication were not included, although the authors did try to cover as many articles as possible in the search. Third, diagnostic value reported in each study was dependent on the radiotracers used, thus results should be interpreted with caution. Fourth, there exists variability among readers to interpret the angiographic images in terms of the degree of stenosis, for example, 50% or more stenosis [52]. Last, this analysis was restricted to English language literature, which could introduce biased opinion to the results. PET/CT has more potential advantages because it integrated two modalities but not many studies were found.

In conclusion, this analysis shows that PET has higher sensitivity, specificity and accuracy for detection of CAD than SPECT and PET/CT. PET can be used as a reliable, less invasive modality for functional analysis of patients suspected of CAD. Further studies comprising a large sample size are needed to verify these results.
